# Cellular Defensive Mechanisms of Tea Polyphenols: Structure-Activity Relationship

**DOI:** 10.3390/ijms22179109

**Published:** 2021-08-24

**Authors:** Van-Long Truong, Woo-Sik Jeong

**Affiliations:** Food and Bio-Industry Research Institute, School of Food Science & Biotechnology, College of Agriculture and Life Sciences, Kyungpook National University, Daegu 41566, Korea; truonglongpro@gmail.com

**Keywords:** cellular antioxidant defense, polyphenols, green tea, black tea, structure-activity relationship

## Abstract

Tea is particularly rich in polyphenols, including catechins and theaflavins, thearubigins, flavonols, and phenolic acids, which are believed to contribute to the health benefits of tea. The health-promoting effects of tea polyphenols are believed to be related to their cellular defensive properties. This review is intended to briefly summarize the relationship between the chemical structures of tea polyphenols and their biological activities. Tea polyphenols appear as direct antioxidants by scavenging reactive oxygen/nitrogen species; chelating transition metals; and inhibiting lipid, protein, and DNA oxidations. They also act directly by suppressing “pro-oxidant” enzymes, inducing endogenous antioxidants, and cooperating with vitamins. Moreover, tea polyphenols regulate cellular signaling transduction pathways, importantly contributing to the prevention of chronic diseases and the promotion of physiological functions. Apparently, the features in the chemical structures of tea polyphenols are closely associated with their antioxidant potentials.

## 1. Introduction

Tea is prepared from the shoot, young leaves, mature leaves, or stems of the tea plant (*Camellia sinensis*), and it is one of the most widely consumed plant-based beverages worldwide. It has been traditionally used in China and several Asian countries since ancient times as a daily drink and folk medicine, with records of its use for the prevention and treatment of various diseases. Recent research has also demonstrated the numerous health benefits of tea, such as antioxidant, anti-inflammatory, and anticancer activities [1,2,3]. It is believed that the potential health benefits associated with tea consumption have been attributed to the antioxidant properties of tea polyphenols [4].

Based on the manufacturing processes and degrees of fermentation, tea can be categorized into green, white, yellow, oolong, black, and dark tea. Each type of tea has its own unique and crucial manufacture process. White tea is prepared by withering and drying unopened tea bubs with fine silvery-white hairs, which give it the name “white tea.” Green tea is produced from buds and young leaves that are rapidly heated and dried to inactivate enzymes and native microflora, which are capable of oxidizing tea polyphenols. The process for yellow tea production is similar to that of green tea production but contains an additional step of heaping (yellowing) for deactivated tea leaves, which provides the characteristic aroma and color of yellow tea. Black tea is manufactured through complete fermentation of tea leaves that are crushed and allowed to undergo enzyme-mediated oxidation, whereas oolong tea is a partially fermented product by limiting the time of oxidation. Dark tea undergoes a postfermentation process (pile fermentation), wherein environmental or special microorganisms modify the chemical composition, flavor, and appearance of tea leaves [4,5].

Fresh tea leaves contain a mixture of compounds that impart an astringent and bitter taste. However, the transformation of original compounds under specific processing conditions such as temperature, humidity, and enzymatic and nonenzymatic oxidations importantly contributes to the formation of the specific taste, flavor, and features of each tea type [5]. Black tea is the most favorite product, which generally accounts for 75–78% of global tea consumption, followed by green tea with 20% and oolong tea with 2%. Other tea types account for insignificant proportions of the world production of tea. Asian countries primarily consume green, yellow, oolong, and dark teas, whereas western countries prefer black tea [4,5].

## 2. Chemical Properties of Tea Polyphenols

**Figure 1:**
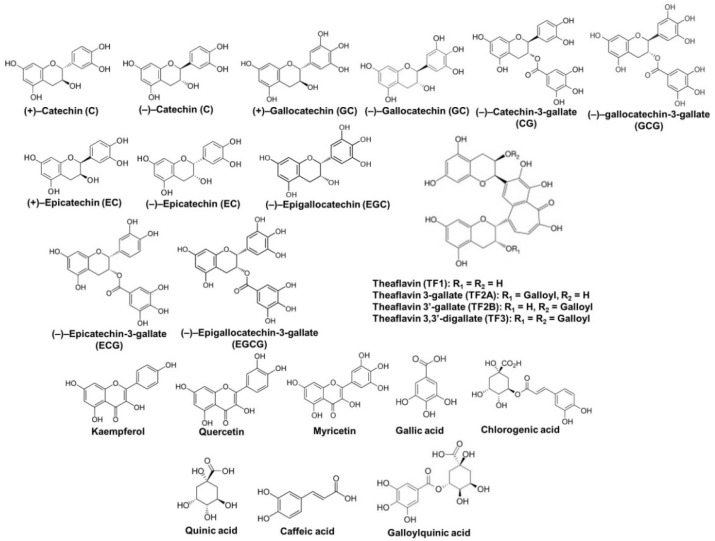
Chemical structures of major tea polyphenols.

Tea contains several groups of polyphenols that include flavan-3-ols and their oligomers, flavonols and their glycosides, phenolic acids and hydrolysable tannins, theaflavins, and thearubigins (Figure 1) [5]. The contents of polyphenols vary in different types of tea depending on the manufacturing procedure. White tea contains the maximum level of polyphenols as it undergoes the least amount of processing. Therefore, white tea has abundant potent antioxidants and may be even more effective than green tea. Oolong, yellow, black, and dark teas contain lower amounts of polyphenols than white and green teas due to the fermentation of tea production that significantly decreases the level of polyphenols. The total polyphenol content accounts for 10–15% of dried green tea weight and 5% of dried black tea weight. The amounts of catechins in green tea are higher than those in oolong, black, and dark teas. Compared with green tea, oolong tea contains approximately half the epigallocatechin-3-gallate (EGCG), whereas the content of polymerized polyphenols is double [4].

Monomeric flavan-3-ols, known as catechins, are the major tea polyphenols. Flavan-3-ols are chemically characterized by two benzene rings referred to as the A-ring (resorcinol moiety) and the B-ring (catechol moiety) and a dihydropyran heterocycle (the C-ring) with a hydroxyl group at carbon 3. Catechin molecules have two chiral centers at the positions of carbons 2 and 3 of the C-ring, and hence each molecule has four diastereoisomers, including two isomers of *trans* configuration and other two of *cis* configuration. The *trans* and *cis* isomers are referred to as catechins (2R,3S and 2S,3R configurations) and epicatechins (2R,3R and 2S,3S configurations), respectively [6]. Structurally, epicatechin has a meta-dihydroxyl group in the A-ring at carbons 5 and 7, an ortho-dihydroxyl group in the B-ring at carbons 3′ and 4′, and a hydroxyl group at carbon 3 in the C-ring. Unlike epicatechin, epigallocatechin has a trihydroxyl group at carbons 3′, 4′, and 5′ in the B-ring, whereas epicatechin-3-gallate has a gallate moiety at carbon 3 in the C-ring. EGCG possesses trihydroxyl groups at carbons 3′, 4′, and 5′ in the B-ring and a gallate moiety located at carbon 3 in the C-ring [6,7]. In general, the 2R,3R configurations of flavan-3-ols, including (−)–epicatechin (EC), (−)–epicatechin-3-gallate (ECG), (−)–epigallocatechin (EGC), and (−)–EGCG, are predominant in tea leaves and tea products. However, the 2S,3R configurations of flavan-3-ols, such as (+)–catechin (C), (−)–catechin-3-gallate (CG), (−)–gallocatechin (GC), and (−)–gallocatechin-3-gallate (GCG), are found in *C. sinensis* tea leaves at extremely low degrees. In addition, several procyanidins, oligomers and polymers of flavan-3-ol units via the C4–C8 bond, have been identified in fresh tea leaves and oolong tea [5,8]. The basic unit of these procyanidins could be either 2R,3R-epicatechins or 2S,3R-catechins. However, high-polymeric procyanidins are easily oxidized during processing because the C4–C8 bonds between flavan-3-ol units are not stable under high temperature and moisture conditions [9]. 

Catechins are easily transformed into various products by enzymatic and nonenzymatic oxidations under the conditions of temperature, humidity, and oxygen during the manufacturing process. The major oxidation products of catechins in black tea are benzotropolone compounds, also known as theaflavins, including theaflavin (TF1), theaflavin-3-gallate (TF2a), theaflavin-3′-gallate (TF2b), and theaflavin-3,3′-digallate (TF3). Theaflavins possess a benzotropolone skeleton that is produced from the oxidative dimerization of hydroxyl groups in the B-ring of appropriate pairs of catechins under the catalysis of polyphenol oxidase [4]. Thearubigins are deep oxidation products of catechins and theaflavins and may be formed by the combination of theaflavins and other oxidation products of catechins. Theaflavins account for 3–5% of black tea solid extract, whereas thearubigins constitute ~20% of black tea solid extract [5].

Flavonols and their glycosides are present in tea leaves and tea products. Flavonols structurally differ from flavan-3-ols in that they have a ketone group at carbon 4 and a C2–C3 double bond in the C-ring, which may contribute to their antioxidant and anti-inflammatory properties [5,10]. Kaempferol, quercetin, and myricetin are predominant flavonol glycones with similar levels in both green and black teas. Diverse flavonol glycosides such as kaempferol-3-O-glucosyl-(1-3)-rhamnosyl-(1-6)-galactoside, quercetin-3-O-glucosyl-(1-3)-rhamnosyl-(1-6)-glucoside and quercetin-3-O-glucosyl-(1-3)-rhamnosyl-(1-6)-galactoside, kaempferol-rhamnodiglucoside, myricetin-glucoside, and quercetin-3-rutinoside have been isolated from fresh tea leaves and tea products [11]. Although flavonols and their glycosides are present in low levels, they importantly contribute to the health benefits of tea polyphenols because flavan-3-ols are oxidized or degraded throughout processing.

Several phenolic acids are present in fresh tea leaves and tea beverages, which can occur individually or be conjugated with one molecule of glycosyl to generate hydrosable tannins. Numerous phenolic acids and their hydrolysable tannins such as gallic, hydroxybenzoic, hydroxycinnamic, quinic, and affeoylquinic acids have been identified in teas [12,13].

## 3. Antioxidant Activity of Tea Polyphenols

Oxidative/nitrosative stress is defined as the excess production of reactive oxygen species (ROS) and/or reactive nitrogen species (RNS) relative to antioxidant systems. ROS and RNS consist of free radicals and nonradicals such as singlet oxygen (^1^O_2_), superoxide anion radical (O_2_•^−^), hydrogen peroxide (H_2_O_2_), hydroxyl radical (•OH), nitric oxide (NO•), peroxynitrite anion (ONOO^−^), peroxyl radical (ROO•), and others. ROS/RNS can be formed from endogenous and exogenous sources. Various types of immune cells and neutrophils also produce ROS/RNS along with the release of pro-inflammatory mediators and cytokines in response to pathogen invasion. ROS/RNS production is also induced by exposure to various environmental stimuli such as pollutants, medicines, xenobiotics, ultraviolet (UV) and ionizing radiation, and metals and metalloids [14,15]. Overproduction of ROS/RNS that causes oxidative/nitrosative stress can attack cellular macromolecules such as lipids, proteins, and nucleic acids, thereby causing the disruption of enzyme activity, alteration of signaling pathways, destruction of cellular membrane, and DNA damages and mutagenesis [16]. There is obvious evidence indicating that ROS/RNS is related to the development of multiple diseases such as inflammatory bowel diseases, atherosclerosis, and cancer [17,18]. Therefore, it is necessary to maintain “redox homeostasis” to protect cells/tissues from oxidative/nitrosative damages, and thus prevent the development of diseases.

**Figure 2:**
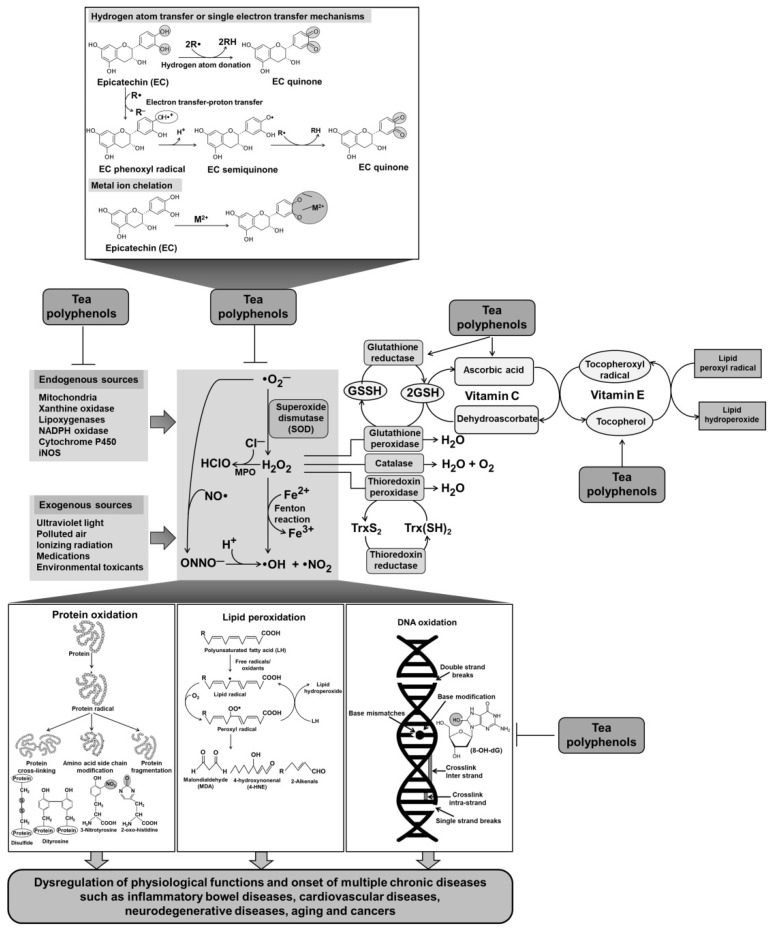
Scheme showing ROS/RNS formation and possible antioxidant mechanisms of tea polyphenols. ROS/RNS can be produced by endogenous sources (e.g., mitochondria, xanthine oxidase, lipoxygenase, NADPH oxidase, cytochrome P450, and inducible nitric oxide synthase) and exogenous sources (e.g., ultraviolet light, polluted air, ionizing radiation, medications, and environmental toxicants). Under normal conditions, ROS/RNS are scavenged and neutralized by intracellular antioxidant defense systems, including enzymatic antioxidants (e.g., superoxide dismutase, catalase, glutathione peroxidase, and thioredoxin peroxidase) and nonenzymatic antioxidants (e.g., vitamin C and vitamin E). However, oxidative/nitrosative stress is associated with an overproduction of ROS/RNS that attacks cellular biomolecules such as proteins, lipids, and DNA, thereby resulting in the dysregulation of normal physiological functions and the onset of multiple chronic disorders such as cardiovascular diseases, inflammatory bowel diseases, and cancers. Tea polyphenols can inhibit ROS/RNS formation, scavenge and neutralize radicals and oxidants, upregulate intracellular antioxidant defense systems, and suppress oxidative/nitrosative biomolecule damages, thereby preventing the development of diseases. MPO, Myeloperoxidase; iNOS, Inducible nitric oxide synthase; O_2_, oxygen; O_2_•^−^, Superoxide anion; H_2_O_2_, Hydrogen peroxide; •OH, Hydroxyl radical; •NO, Nitric oxide; •NO_2_, Nitrogen dioxide; ONOO^−^, Peroxynitrite; HClO, Hypochlorous acid; GSH, Glutathione; GSSG, Glutathione disulfide; Trx, Thioredoxin.

Plant cells synthesize polyphenolic compounds as a defense mechanism in response to environmental stressors such as microbial invasion. It is expected that polyphenols exhibit similar protective effects in the human body after the consumption of diet plants containing these compounds. Tea polyphenols are recognized as strong antioxidants that act through several mechanisms such as neutralization of radicals, chelation of transition metals, inhibition of pro-oxidative enzymes, and induction of intracellular antioxidant enzymes [1,19]. Green tea has a higher antioxidant capacity than oolong tea and black tea, and the total antioxidant potential is strongly related to the total polyphenolic content of tea [4]. In this review, we discuss the antioxidant mechanisms of tea polyphenols and the relevant evidence (Figure 2).

### 3.1. Direct Antioxidant Activity of Tea Polyphenols

Tea polyphenols are efficient primary antioxidants; however, the radical scavenging activity, metal-chelating potential, and reducing power vary according to their structural features. Tea polyphenols consist of a large number of phenolic structural units that have different redox potentials. The number and substitution positions of the phenolic units provide them with specific physiochemical properties. In addition, the nature of substituents, degree of polymerization, and extent of glycosylation affect the antioxidant activity of tea polyphenols [20]. An increased number of hydroxyl aromatic rings may exert a higher antioxidant activity. A comparative study has shown that the scavenging ability of flavonoids with an ortho-5,6-dihydroxyl group in the A-ring is much stronger than that of flavonoids with a meta-5,7-dihydroxyl group in the A-ring. Furthermore, the antiradical activity of flavonoids with an ortho-3′,4′-dihydroxyl group in the B-ring is more effective compared with flavonoids with only a meta-5,7-dihydroxyl group in the A-ring [21]. In general, several structural features such as an ortho-3′,4′-dihydroxyl (catechol) group or a 3′4′5′-trihydroxyl (gallate) group in the B-ring, a hydroxyl group or a gallate group esterified at the carbon 3 position in the C-ring, and a meta-5,7-dihydroxyl group in the A-ring are primarily responsible for the antioxidant capacity of tea catechins [22].

The mode of the antioxidant activity of polyphenols can be based on either hydrogen atom transfer or single electron transfer or both. Tea polyphenols easily scavenge radicals and oxidants by donating hydrogen atoms from their hydroxyl groups or by electron transfer–proton transfer, forming less reactive phenoxyl radicals. By rearranging the unpaired electron on the aromatic core, phenoxyl radicals transform into resonance structures—quinones—with low activity [1]. In catechin molecules, the catechol B-ring (3′4′-dihydroxyl group) is more easily oxidizable compared with the resorcinol A-ring [23]. The electron-donating ability of tea polyphenols also reflects the transition metal-reducing power of these biomolecules. Tea polyphenols can reduce transition metal ions such as iron and copper to break the radical chain reaction. Hydroxyl groups in the B-ring and the 3-hydroxyl group in the C-ring are important structural features responsible for the reducing capacity of catechins [24].

#### 3.1.1. Radical and Oxidant Scavenging Activities of Tea Polyphenols

Tea polyphenols were found to scavenge various types of reactive oxygen, nitrogen, and chlorine species in vitro, including superoxide anions, singlet oxygen, hydroxyl radicals, hydrogen peroxide, nitric oxide, peroxyl radicals, hypochlorous acid, and peroxynitrous acid [22,25,26]. The in vitro scavenging and/or antioxidant activity of a certain substance is generally evaluated using chemical-based assays. The ferric-reducing antioxidant power (FRAP) assay that was used to detect the total antioxidant power of freshly prepared infusions of 25 types of tea demonstrated that green tea (FRAP values ranging 272–1144 µmol/g) exerted more antioxidant potential in average compared with oolong (233–532 µmol/g) and black teas (132–654 µmol/g), and the antioxidant capacity was strongly related to the total phenolic content of the tea [27]. Likewise, a positive correlation also exists between the total polyphenol contents of tea and the antioxidant capacity measured by other methods, including oxygen radical absorbance capacity assay, copper-reducing power, DPPH scavenging, and superoxide scavenging [28,29]. Another study investigated the phenolic profiles and antioxidant activities of 30 tea infusions from green, black, oolong, white, yellow, and dark teas. The results showed that in general, green tea exhibited the highest antioxidant capacity and total phenolic content was determined by FRAP, Trolox equivalent antioxidant capacity, and total phenolic content assays [30]. Likewise, green tea also demonstrated higher phenolic and flavonoid contents as well as higher free radical scavenging and total antioxidant activities than three other black tea varieties in Bangladesh [31].

Tea catechins, the components of tea polyphenols, also exert scavenging activity on various free radicals and oxidants such as superoxide anions, singlet oxygen, hydroxyl radicals, lipid peroxyl radicals, DPPH radicals, and ABTS radical cations [32]. The DPPH radical and superoxide radical scavenging abilities of tea catechins at a final concentration of 10 µM were in the sequence of EGCG > ECG > EGC > EC [33]. Likewise, another study comparing the activities of seven individual catechins (used at concentrations between 0.5 and 10 µM) from green tea tannin mixture indicated that EGCG, GCG, and ECG possessed higher superoxide radical scavenging activities than EGC, GC, EC, and C, suggesting the significance of gallic acid in the structure of catechins [26]. Among the gallate-free catechins, EGC and GC were found to be more effective in superoxide radical scavenging compared with EC and C, implying that a hydroxyl group at the position of carbon 5′ in the B-ring is a vital determinant of such activity. However, these structures did not play a central role in the NO scavenging activity [26].

A study that compared the in vitro antioxidant capacity of typical polyphenols in black tea demonstrated that the hydroxyl radical and DPPH radical scavenging abilities of these compounds were in the order of theaflavins > theabrownins > thearubigins with IC_50_ values of 5.2, 11.4, and 16.8 µg/mL for hydroxyl radical, and 8.1, 18.0, and 36.1 µg/mL for DPPH radical, respectively [34]. The DPPH radical scavenging activity of black tea theaflavins at a concentration of 10 µM was in the order of TF3 > TF2 > TF1, whereas the superoxide radical scavenging ability of theaflavins was similar [33]. Another study evaluated the antioxidant capacities of black tea theaflavins and their gallate esters, namely TF1-, theaflavin-3(3′)-gallate (TF2), and TF3, by comparing with the antioxidant activity of a green tea polyphenol EGCG. The results demonstrated that the order of hydroxyl radical scavenging ability was TF3 > TF2 > TF1 > EGCG with IC_50_ values of 1.6, 3.1, 3.9, and 4.2 μM, respectively, whereas the order of DPPH scavenging activity was TF3 > TF2 > EGCG > TF1 with IC_50_ values of 7.7, 10.9, 12.1, and 33.2 μM, respectively [35]. Theaflavins, including TF1, TF2, and TF3, have two A-rings of flavanols connected by a fused seven-member ring (benzotropolone moiety) with multiple hydroxyl groups, which are considered as a determinant factor for the radical scavenging activity. These structural features may provide more interaction sites with radicals/oxidants compared with catechins. Moreover, TF3 molecule has two gallic acid moieties, and studies have shown that the gallic acid moiety plays a crucial role in the antioxidant activity of tea catechins and theaflavins [32,36,37]. It is remarkable that the benzotropolone moiety may contribute to the antioxidant capacity of theaflavins through the mode of electron donation because of the existence of resonance forms [35].

In addition, all individual tea polyphenols, including TF1, TF2, TF3, and EGCG at concentrations between 0.4 and 3.2 μM, were found to significantly suppress intracellular ROS accumulation in H_2_O_2_-treated HPF-1 cells, and TF1, TF2, and TF3 were found to be more effective in protection against H_2_O_2_-induced HPF-1 cell death compared with EGCG [35]. Interestingly, the effective antioxidant concentrations of TF1, TF2, TF3, and EGCG in cell-based assays are lower than IC_50_ values of these compounds in chemical-based assays, suggesting that tea polyphenols may exert other mechanisms responsible for the protective effects against oxidative cell damage, rather than only scavenging already formed ROS. Green tea polyphenols have been reported to inhibit the 6-hydroxydopamine-induced production of ROS and RNS, thereby protecting SH-SY5Y cells against apoptosis caused by neurotoxin [38]. The free radical scavenging activity of EGCG in vivo (EGCG 40 mg/kg/day was orally administered 90 min before administration of sodium fluoride 25 mg/kg/day for 4 weeks) was observed, as evident from the decreased levels of fluoride-induced superoxide radicals, hydrogen peroxide, and hydroxyl radicals in rat lung tissues [39].

The antioxidant capacity of tea polyphenols was further demonstrated in clinical studies, which reported an increased total antioxidant capacity in the plasma of healthy volunteers after the consumption of tea polyphenols [40,41]. A total of 10 healthy subjects consumed 150, 300, and 450 mL of green tea infusions (2.5, 5, and 7.5 g of dried green tea leaves), and the total antioxidant capacity of plasma was measured by the ABTS^+^ radical scavenging assay at baseline and 1 and 2 h after ingestion. The results demonstrated that the total antioxidant capacity of human plasma was significantly increased after 1 and 2 h of consuming green tea in the amounts of 300 and 450 mL [42]. In another study, the consumption of green tea (400 mL of tea infusion) rapidly increased the plasma antioxidant power, as evaluated by the FRAP assay, with a peak of 4% at 40 min after ingestion in healthy adults; however, the excretion of green tea polyphenols was rapid, with peaks of urinary FRAP values and urinary total polyphenol contents observed at 60–90 min [43].

#### 3.1.2. Inhibition of Lipid Peroxidation by Tea Polyphenols

Lipid peroxidation indicates the oxidative deterioration of lipids containing any number of carbon–carbon double bonds, such as unsaturated fatty acids, phospholipids, glycolipids, and cholesterol. It leads to structural degeneration of the membrane and induces apoptosis and further damage to other cellular macromolecules such as protein and DNA. Lipid peroxidation is a chain reaction, including initiation, propagation, and termination. Various oxidants can attack polyunsaturated fatty acids, which contain multiple double bonds and a methylene group with reactive hydrogen atoms, and thus initiate radical chain reactions [44,45]. Lipid peroxidation can also be driven by factors other than ROS/RNS, such as transition metal ions (Fe^2+^ and Cu^2+^) and enzymes (cytochrome P450s, cyclooxygenases, and lipoxygenases). Various types of aldehydes, ketones, alkanes, carboxylic acids, and polymerization products are formed during the peroxidation process of lipids; they serve as biomarkers of lipid peroxidation. The aldehydes produced via lipid peroxidation such as malondialdehyde (MDA) are highly stable and easily diffuse across the membrane to cross-link or modify the structures of proteins, lipids, DNA, and carbohydrates, thereby resulting in mutagenesis, carcinogenesis, and functional loss and consequently initiating pathogenic conditions [16,46].

Antioxidant compounds can directly scavenge ROS/RNS and chelate transition metal ions to prevent the initiation of lipid peroxidation. The chain-breaking antioxidants also quench peroxide radicals to terminate the chain reaction. A study showed that green tea catechins, used at 30–200 mg/kg sample, concentration-dependently inhibited the oxidation of meat lipids in the order of EGCG ≈ ECG > EGC > EC with highest at 200 mg/kg, and catechins were generally more effective compared with α-tocopherol at the same concentration [47]. In another study, individual green tea polyphenols, including EC, EGC, ECG, and EGCG at concentrations of 0.005–0.5 mM, were found to exert protective effects against iron-induced lipid peroxidation in synaptosomes in the order of EGCG > ECG > EGC > EC [48]. A study that compared the antioxidant potential of four green teas and seven black teas found that all tea extracts inhibited in vitro Cu^2+^-induced lipoprotein oxidation in human serum to a similar extent [49]. Another research reported that theaflavins (at a concentration of 5 µM) present in black tea could exert antioxidant potency as much as catechins in green tea at the same concentration, regarding the inhibition of Cu^2+^-mediated low-density lipoprotein (LDL) oxidation [50]. Using the Rancimat method, it was found that theaflavins exhibited lower inhibition activity on lipid oxidation than tea catechins [33]. In contrast, pretreatment of macrophages and endothelial cells with TF3 decreased the oxidation of cell-based LDL in dose- and time-dependent manners, and the inhibitory effect of tea polyphenols on the cell-mediated LDL oxidation was in the order of TF3 > TF1 ≥ EGCG > EGC > gallic acid [51]. The inconsistent results of these studies may be attributed to the use of different models and experimental methods.

Treatment with tea catechins at concentrations of 2.5–25 µM was found to effectively inhibit lipid peroxidation in HepG2 cells in the order of EGCG > EGC ≥ ECG > EC [52]. Another study showed that pretreatment of PC12 cells with black tea theaflavins (5, 10, and 20 µM) significantly decreased H_2_O_2_-induced ROS production and lipid peroxidation [53]. Likewise, black tea theaflavins at final concentrations of 0.1–10 µM were found to inhibit oxidative stress-induced lipid peroxidation in rat erythrocytes [54]. Moreover, green tea catechins (4 µM) were found to exert a protective effect against free radical-initiated lipid peroxidation in rat liver microsomes, with EC and ECG with ortho-dihydroxyl functionality being especially better antioxidants for microsomal peroxidation [55].

Consumption of green tea solution (3 g/L) for 5 weeks was found to significantly reduce lipid peroxidation, as evident from the decreased levels of lipid hydroperoxides (LOOH), 4-hydroxynonenal (4-HNE), and MDA, and concurrently increase the total antioxidant status in the liver, blood serum, and central nervous tissues of young healthy rats [56]. In a rat model of Parkinson’s disease, it was observed that green tea polyphenols (150 or 450 mg/kg/day-fed for 7 days) attenuated 6-OHDA-induced lipid peroxidation in the midbrain and striatum by preventing ROS/RNS production and preserving free radical scavenging capacity. In particular, green tea polyphenols also reduced the levels of lipid peroxidation in the midbrain and striatum of unlesioned groups without 6-OHDA stimulation [57]. Moreover, pre-administration of EGCG (50 or 75 mg/kg body weight) for 3 consecutive days was found to reduce CCl_4_-induced liver injury in association with lower levels of lipid peroxidation and nitric oxide-generated mediators [58].

A clinical study on 34 Portuguese subjects demonstrated that regular consumption of green tea (1 L daily for 4 weeks) significantly reduced the serum levels of lipid peroxidation products, MDA, and 4-HNE, and simultaneously increased the total antioxidant status [59]. Green tea intervention (3 cups/day for 3 months) also significantly improved the plasma antioxidant status and reduced plasma lipid peroxidation in patients with Parkinson’s disease [60].

#### 3.1.3. Inhibition of DNA Oxidation by Tea Polyphenols

Highly reactive oxidants can directly attack DNA, leading to oxidative DNA damage such as base modification, deoxyribose damage, single- and/or double-strand breakage, and DNA crosslinking. Moreover, RNS reacts with DNA and causes nitrative DNA damage due to the formation of 8-nitroguanine [46]. Cell death and mutation originating from DNA lesions have been implicated in IBD, cancer, and aging [61].

In addition to the direct scavenging effect of free radicals before they react with DNA, a study reported that tea catechins, including EC, EGC, ECG, and EGCG, at low concentrations (*ca.* micromolar) can alleviate hydroxyl radical-induced DNA single-strand breaks and base damage via rapid chemical repair of DNA radicals. That study also described the mechanism of hydrogen atom transfer and/or electron transfer from catechins to radical sites on DNA [62]. Likewise, black tea theaflavins (20 or 50 µM) have been shown to prevent oxidative stress-induced DNA damage in rat normal liver epithelial RL-34 cells, as evaluated using 8-hydroxydeoxyguanosine (8-OHdG) levels and comet assays [63]. Another study showed that total body irradiation (TBI)-induced oxidative stress and oxidative DNA damage, as analyzed using the levels of 2,7-dichlorodihydrofluorescein diacetate, MitoSox, dihydroethidium, and 8-oxoguanine, were decreased in mouse hematopoietic stem cells by treatment with theaflavins (administered at a dose of 50 mg/kg/day 1 day before TBI and up to 7 days after TBI) [64]. Several green tea polyphenolic compounds such as tannic acid, gallic acid, and ellagic acid at a concentration of 10 µg/mL have been found to effectively suppress H_2_O_2_-induced intracellular ROS accumulation as well as lipid peroxidation and oxidative DNA damage in human lung fibroblast (IMR-90) cells [65]. Dietary administration of 0.05% Polyphenon-B (a mixture of black tea polyphenols) or 0.05% BTF-35 (a well-characterized black tea extract enriched with theaflavins and catechins) was found to prevent oxidative DNA damage, as indicated by the reduced formation of 8-OHdG adducts in a model of 7,12-dimethylbenz[a]anthracene (DMBA)-induced hamster buccal pouch carcinogenesis [66]. Oral administration of a dose of green tea polyphenol extract equivalent to the human dose of 500 mL green tea/day for 5 days was found to protect lymphocytes and, to a lesser extent, inner organs such as colonocytes and hepatocytes against oxidative DNA damage in rats [67].

Clinical studies have shown that high consumption (4 cups/day) of decaffeinated green tea but not decaffeinated black tea significantly decreased the urinary level of 8-OHdG, a marker of oxidative DNA damage, by ~31% in smokers after a 4-month intervention [68]. In particular, green tea intervention (4 cups/day for 4 months) may be more effective in the subgroup of smokers who are positive to glutathione S-transferase (GSTM1 and/or GSTT1) [69]. These data suggest that regular consumption of green tea exerts protective effects against cigarette smoking-induced oxidative DNA damage.

#### 3.1.4. Inhibition of Protein Oxidation by Tea Polyphenols

In addition to lipid peroxidation and oxidative DNA damage, free radicals and oxidants cause protein modification by the oxidation of amino acid side chains, the formation of protein–protein cross-linkages, and the oxidation of protein backbone, which result in protein fragmentation. Amino acid residues are among the preferred targets for oxidant attack. ROS-mediated oxidation of lysine, arginine, proline, and threonine residues in protein may lead to the formation of protein carbonyls (PCO), a well-known marker of protein oxidation by ROS [70]. In addition, RNS and chloride radicals can attack proteins by nitration or chloration of amino acid side chains (e.g., nitrotyrosine and chlorotyrosine), which are found in various pathological conditions, including IBD [44]. Protein oxidation and/or nitration can cause modifications in their structure and function, consequently resulting in abnormalities in cellular signaling pathways, cytoskeletons, and others, and these phenomena have been observed in various chronic diseases.

It has been reported that green tea polyphenols can inhibit chronic UVB-induced protein oxidation in HS68 human skin fibroblast cells and mouse skin tissues [71]. Individual tea catechins, including EC, EGC, and EGCG, could inhibit tryptophan oxidation and protein carbonylation of both human serum albumin and human hemoglobin under-stimulated hyperglycemic conditions [72]. Consumption of green tea extract rich in catechins was found to decrease protein oxidation, as evident from the reduced level of PCO in the hippocampal tissue of aged rats [73] and in the plasma of patients with Parkinson’s disease [60].

Tyrosine is exceptionally susceptible to RNS such as peroxynitrite, forming 3-nitrotyrosine [74]. It has been reported that tea polyphenols exert protective effects against peroxynitrite-dependent nitration reactions by competitively inhibiting the nitration of tyrosine. The ability of individual tea polyphenols (10 µM) to prevent peroxynitrite-induced tyrosine nitration was in the sequence of ECG ≈ EGCG ≈ gallic acid > catechin ≈ epicatechin ≈ EGC, and all polyphenols exhibited more efficacy than the equivalent concentration of trolox [75]. Exposure of SY5Y cells to 6-hydroxydopamine was found to increase the level of protein-bound 3-nitrotyrosine, which was dose-dependently suppressed by pretreatment with green tea polyphenols (50–200 µM) [38]. Pretreatment of IRC mice with EGCG (50 or 75 mg/kg for 3 consecutive days) also prevented the formation of carbon tetrachloride (CCl_4_)-induced protein-bound 3-nitrotyrosine [58]. Likewise, treatment with green tea polyphenols significantly decreased the level of protein-bound 3-nitrotyrosine in the midbrain and striatum of the 6-OHDA-induced rat model of Parkinson’s disease [57] and in the colon of dinitrobenzene sulfonic acid (DNBS)-induced mouse model of colitis [76], which could account for its ability to neutralize ROS/RNS.

#### 3.1.5. Metal Chelation by Tea Polyphenols

Some of the transition metal ions participate in certain oxidation reactions in the body and produce a large number of free radicals. It is believed that transition metal ions such as Fe^2+^ and Cu^2+^ catalyze the production of highly reactive oxidant species, resulting in lipid peroxidation and DNA and protein damages. Fe^2+^, even in minute amounts, reacts with hydrogen peroxide to generate hydroxyl radical by redox cycling, known as the Fenton reaction [44]. Unlike iron, the reaction between Cu^2+^ and H_2_O_2_ is more intricate, which has been proposed to occur through two major alternative mechanisms, including “free radical” and “complex” reaction mechanisms. In the “free radical” mechanism, Cu^2+^ oxidizes H_2_O_2_ to superoxide radical, with Cu^+^ being formed in this process that can react with excess H_2_O_2_ to form hydroxyl radicals (Fenton-like reaction). Cu^2+^ also forms a complex with peroxide without the formation of radical species through the “complex” mechanism [77]. Therefore, chelation of metal ions by antioxidant compounds may abolish their pro-oxidant ability and prevent metal-mediated oxidation.

Tea polyphenols have been indicated to chelate metal ions to form inactive complexes and prevent the generation of potential free radicals, thereby attenuating metal-induced oxidation reactions. It has been suggested that 3′,4′-dihydroxy catechol in the B-ring and the gallate moiety in tea flavonoids are responsible for the chelation of transition metals [78]. Green tea catechins, including EC, EGC, ECG, and EGCG, were found to inhibit metal-induced lipid peroxidation in cultured rat hepatocytes and synaptosomes by chelating ferrous/ferric, copper, and vanadium ions, in which iron-chelating activity of individual catechin (at 0.5 mM each) was in the order of EGC > ECG = EGCG > EC [48,79]. Spectrophotometric titration analysis showed that polyphenols extracted from both green and black teas at the final concentrations of 10–60 µg/mL could sequester ferric ion to form a redox-inactive ferric–polyphenol complex, and that green tea and black tea had a similar iron-chelating potential [80]. Another study demonstrated the binding abilities of black tea theaflavins such as TF1, TF2a, TF2b, and TF3 at a concentration of 40 µM to iron and copper ions using spectroscopic analysis [37]. Digallate ester (TF3) exerted a higher iron-chelating activity than monogallates, whereas the interactions with copper were almost similar for all four theaflavins. That study suggested that the gallate moiety of theaflavins was primarily responsible for iron chelation, whereas copper could interact with the hydroxyl groups on the polyphenolic rings of basic theaflavin molecule rather than with the gallate group [37]. This has been supported by evidence indicating that phenolic acids, including gallic acid, have a strong binding ability to iron, in contrast to the minimal formation of the copper–gallate complex [37,81,82]. In a study comparing the metal-chelating activity of different tea infusions, it was observed that green teas exhibited the lowest metal-chelating ability, whereas white tea and black orthodox tea exhibited the highest activity. This finding implies that components of other catechins could contribute to the chelating capacity [83].

### 3.2. Indirect Antioxidant Activity of Tea Polyphenols

#### 3.2.1. Inhibition of “Pro-Oxidant” Enzymes by Tea Polyphenols

Along with the direct antioxidant capacity, tea polyphenols have been shown to inhibit radical and oxidant production by regulating different types of oxidase enzymes. It has been reported that tea polyphenols suppress the activity of nicotinamide adenine dinucleotide phosphate (NADPH) oxidase by downregulating the expressions of NADPH oxidase subunits (p22phox and p67phox), thereby reducing ROS production in high glucose-induced bovine aortic endothelial cells [84]. An upregulated expression of NOX4, a pro-oxidant enzyme, was detected in the lineage-c-kit+ cells of mice treated with ionizing radiation, which was attenuated by theaflavin administration at a dose of 50 mg/kg/day [64]. In an in vitro study, green tea extract and its pure compounds, (+)-catechin and EGCG, (1000 ppm) were found to inhibit the activity of xanthine oxidase, with EGCG exhibiting the most potent efficacy [85]. Five tea polyphenols, viz., EGCG, gallic acid, TF1, TF2, and TF3, were found to exert inhibitory effects on xanthine oxidase activity and intracellular ROS production in HL-60 cells, in which TF3 is the most potent competitive inhibitor of xanthine oxidase with IC50 value of 4.5 µM. The results of that study suggest that in addition to the direct scavenging of species, tea polyphenols act as inhibitors of xanthine oxidase that contributes to ROS formation [86].

Inducible nitric oxide synthase (iNOS) is responsible for the production of large amounts of NO radicals, which rapidly react with superoxide radicals to form ONOO^−^ and other NO-derived oxidants that are capable of damaging cellular macromolecules. In cultured macrophages, individual catechins and theaflavins extracted from green and black teas blocked lipopolysaccharide (LPS)-induced iNOS gene expression and iNOS activity [87,88,89]. Compared with a positive control omeprazole, oral administration of black tea extract (40 mg/kg) or theaflavins (1 mg/kg) for 3 days were found to be more effective in inhibiting the activity and expression of iNOS as well as the production of NO in mice with gastric ulcer. Moreover, the activity of myeloperoxidase (MPO), which is responsible for producing hypochlorous acid during neutrophil respiratory burst, was reduced in gastric ulcerated mice upon supplementation with black tea extract or theaflavins [90].

Lipoxygenases (LOX) and cyclooxygenases (COX) contribute to the formation of oxidants through their peroxidase activity. Purified individual catechins, including EGCG, EGC, and ECG, and theaflavins extracted from black tea at a concentration of 30 µg/mL have been reported to suppress the activities of COX-2 and 5-, 12-, and 15-LOX in human normal colon mucosa and colon tumors [91]. Consumption of 0.2% green tea polyphenols in water for 30 days was found to significantly reduce UVB-induced epidermal COX activity in SHK-1 hairless mice [92], and topical pre-application of polyphenols extracted from green tea or black tea also resulted in significant inhibitions of 12-O-tetradecanoylphorbol-13-acetate (TPA)-induced epidermal COX and LOX activities [93,94].

#### 3.2.2. Induction of Intracellular Antioxidant Defense System by Tea Polyphenols

Cellular redox homeostasis is ensured by a complex endogenous antioxidant defense system, including enzymatic and nonenzymatic antioxidants. Nonenzymatic antioxidants consist of thiols, metal-binding proteins (transferrin, ferritin, lactoferrin, ceruloplasmin, and even albumin), uric acid, coenzyme Q, bilirubin, melatonin, and lipoic acid [95]. Among these, tripeptide γ-glutamylcysteinylglycine or glutathione, abundantly present in all cell types at millimolar concentration, is the major nonenzymatic antioxidant of the cell defense system. Glutathione exists in either reduced (GSH) or oxidized (GSSG) form, and the ratio of GSH/GSSG is a good index of oxidative stress of a living system [96]. Depletion of glutathione results in an impaired antioxidant defense in cells/tissues, causing an imbalance of redox homeostasis and consequently facilitating various pathological states [46].

Recent research has indicated a close correlation between endogenous antioxidants and dietary polyphenols, wherein polyphenolic compounds can counteract the decrease in glutathione concentration under the condition of oxidative stress [97]. In the case of lead-induced toxicity in PC12 cells, both EC and ECG (100 µM) remarkably restored the GSH/GSSG ratio and glutathione reductase (GR) activity, and the galloylated catechins ECG and EGCG (100 µM) increased the protein sulfhydryl groups (PSH)/glutathione−protein mixed disulfide (GSSP). In contrast, EGC (100 µM) significantly reduced the GSH/GSSG and PSH/GSSP ratios as well as the GR activity in the lead-treated PC12 cells. These data suggest that tea catechins exert distinct effects on the status of intracellular thiols, possibly being associated with chemical structures and/or regulation of varied gene expression [98]. It was also observed that green tea polyphenols restored the GSH content in the cerebral cortex and hippocampus of rats with ischemia–reperfusion [99] and in mice with acetaminophen-induced liver toxicity [100,101]. EGCG (50 µM), the major and most active component in tea polyphenols, has been reported to inhibit the proliferation of activated hepatic stellate cells by increasing the de novo biosynthesis of GSH through the upregulation of glutamate–cysteine ligase expression [102]. Supplementation of green tea beverage (4 cups/days) and green tea extract (2 capsules/day) for 8 weeks was found to significantly increase the plasma antioxidant capacity and whole blood glutathione activity in obese patients with metabolic syndrome [103]. In a clinical trial on the photoprotective effects of a green tea polyphenol, topical application of EGCG was found to protect human skin from UV-induced glutathione depletion [104].

Endogenous antioxidant enzymes, including catalase (CAT), glutathione peroxidase (GPx), and superoxide dismutase (SOD), function as a first line of defense against a massive attack of oxidants. Accumulating studies have indicated that consumption or supplementation of tea polyphenols can recover redox homeostasis by enhancing the activity of endogenous antioxidant enzymes. EGCG (50 µg/mL) treatment was found to protect human dermal fibroblasts from H_2_O_2_-induced oxidative damage by increasing the activity of GPx and SOD and by decreasing the levels of MDA [105]. Likewise, black tea theaflavins, including TF1, TF2a, TF2b, and TF3 at concentrations of 5–20 µM, exhibited neuroprotective effects against oxidative stress-stimulated apoptosis in PC12 cells by enhancing the activities of SOD and CAT [53].

Several studies have demonstrated the positive effects of tea polyphenols on redox homeostasis by enhancing the levels of intracellular antioxidants such as GSH, CAT, GPx, and SOD, thereby inhibiting lipid peroxidation in several animal models [106,107,108]. These data suggest that tea polyphenols improve the body’s antioxidant defense potential and regulate the oxidoreductase system [1]. A study examining the protective effects of catechins (2% catechin in diet for either 2 weeks or 4 weeks prior to UVB irradiation) against UVB-induced mouse skin damage reported that dietary supplementation with catechins could activate the cellular antioxidant defense system by regulating the expression of several antioxidant enzymes [109]. Another study reported that oral administration of tea polyphenols (400 mg/kg) counteracted oxidative stress and intestinal damage in C57BL/6 mice with bacterial infection by inhibiting myeloperoxidase activity and MDA production and by enhancing the activity of several intestinal antioxidant enzymes [110]. In a rat model of azathioprine-induced liver injury, it was observed that oral pre-administration of green tea polyphenols (100 or 300 mg/kg) prevented the elevation of liver lipid peroxidation and protein carbonyl content as well as the depletion of hepatic total antioxidant activity, including GSH, CAT, and GPx [111]. In another study, administration of green tea containing tannins was found to protect Sprague Dawley rats from arsenic-induced hepatic and renal oxidative injury by increasing the levels of GSH, GPx, and SOD and concurrently reducing the levels of lipid peroxidation and nitrite/nitrate. Moreover, the tannin-rich fraction of green tea exhibited a greater antioxidant activity than the detannified fraction, indicating that tannins at least partially contribute to the indirect antioxidant property of tea polyphenols [112]. EGCG was also found to exert similar protective effects against arsenic-induced hepatotoxicity in rats [113].

Although tea polyphenols are considered to be powerful antioxidants, which can scavenge oxidants/free radicals and chelate transition metals, accumulating pieces of evidence have indicated that tea polyphenol can also act as pro-oxidants, both in vitro and in vivo. Tea polyphenols are unstable and can undergo auto-oxidative process in certain cellular environments, leading to the production of ROS [19]. It was found that tea catechins at concentrations less than 10 µM exerted protective effects against oxidative damage, while tea catechins at concentrations higher than 10 µM provoked the production of hydrogen peroxide [114]. Pro-oxidant effects of tea polyphenols contribute to, in part, inhibition of cancer cell viability and induction of tumor cell apoptosis [115]. More recent studies have showed that pro-oxidant properties of polyphenols also trigger an adaptive response that stimulates endogenous antioxidant defense systems capable of preventing against oxidative stress-mediated pathological conditions [115,116]. Increased intracellular level of GSH in human erythrocytes by polyphenols was found to be associated with a slight increase in ROS [116]. Green tea polyphenol EGCG at low concentrations (less than 50 µM) apparently induced mild oxidative stress, consequently activating cellular antioxidant defense, as evident from enhanced GSH synthesis and increased GST activity, whereas EGCG at higher levels (over 200 µM) persistently elevated intracellular ROS that overwhelms antioxidant defense system, leading to oxidative damage [117]. Black tea polyphenol TF3 at a nontoxic concentration of 250 µM induced oxidative stress and subsequently stimulated resynthesis of intracellular GSH in GN46 fibroblasts [118]. Therefore, potential health benefits of tea polyphenols may be partially attributed to their pro-oxidant properties that provoke low levels of ROS and thereby boost antioxidant defense systems to counteract oxidative stress [119].

#### 3.2.3. Antioxidant Synergisms of Tea Polyphenols and Vitamins

Vitamins play an essential role in maintaining normal physiological functions such as metabolism, body growth, and development. Several vitamins are also involved in the antioxidant defense system of the body. It has been reported that tea polyphenols could interact with α-tocopherol (vitamin E) to synergistically enhance their antioxidant activity in various systems. Evaluation of the free radical-induced peroxidation of linoleic acid disclosed that tea polyphenols in combination with α-tocopherol exhibited synergistic antioxidant efficacy in a homogenous solution [120] and in micelles [121] compared with individual compounds. A similar effect was observed on free radical-initiated and benzophenone-photosensitized human LDL peroxidation [122]. These observations have been supported by evidence indicating that in addition to the quenching-initiating and/or -propagating effect of peroxyl radicals, typical green tea polyphenols such as EC, EGC, ECG, EGCG, and gallic acid could effectively reduce the α-tocopheroxyl radical to regenerate α-tocopherol [123,124]. Moreover, although all individual tea polyphenols, vitamin E, and vitamin C are good antioxidants, a mixture of green tea polyphenols, vitamin E, and vitamin C could act synergistically to inhibit free radical-induced lipid peroxidation. Such synergistic efficacy was due to the regeneration of vitamin E by the green tea polyphenol and the regeneration of the latter by vitamin C [125].

## 4. Cellular Defensive Mechanisms of Tea Polyphenols through the Regulation of Signaling Pathways

### 4.1. Regulation of Phase I and Phase II Enzymes by Tea Polyphenols

**Figure 3:**
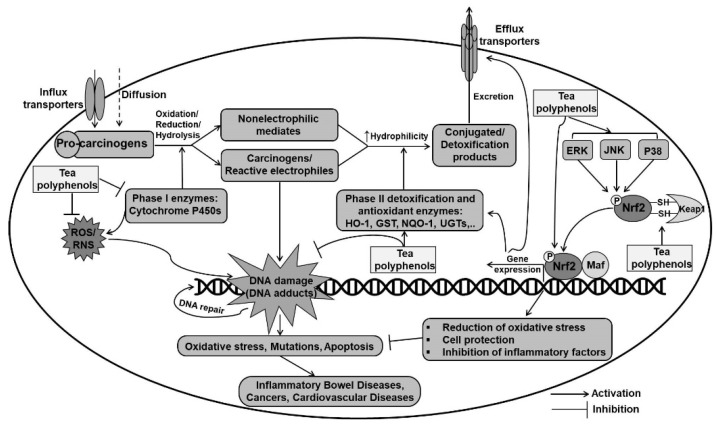
Cytoprotective mechanisms of tea polyphenols via regulation of phase I and phase II enzymes. Keap1, Kelch-like ECH-associated protein 1; maf, Musculoaponeurotic fibrosarcoma; Nrf2, Nuclear factor erythroid 2-related factor 2; HO-1, Heme oxygenase-1; NQO1, NADPH:quinone oxidoreductase 1; GST, Glutathione transferase; ERK, Extracellular signal-regulated kinase; JNK, c-Jun N-terminal kinase; UGTs, UDP-glucuronosyltransferases.

All dietary and environmental carcinogens subjected to the human body undergo metabolism, detoxification, and elimination processes. Cytochrome P450 enzymes (CYPs) are primarily responsible for phase I metabolism, which mainly oxidize chemical molecules to more hydrophilic compounds. Hence, procarcinogens are generally transformed to highly reactive intermediates that can attack cellular molecules such as DNA, lipids, and proteins and trigger the formation of ROS/RNS. The second group of enzymes, known as phase II enzymes, is involved in the detoxification of reactive intermediates and toxicants by catalyzing the conjugation reaction of intermediates with endogenous cofactors, thereby resulting in the production of water-soluble compounds that can be easily excreted in the bile or urine (Figure 3) [126].

Cytoprotective phase II detoxifying and antioxidant enzymes such as heme oxygenase-1 (HO-1), NAD(P)H:quinone oxidoreductase (NQO1), and glutathione-related enzymes importantly contribute to intracellular redox homeostasis. These enzymes are a component of the endogenous defense system that is involved in the neutralization and detoxification of ROS/RNS, xenobiotics, and noxious toxicants from cells before they can damage biomolecules, and thus protects against carcinogenesis and inflammatory diseases. Nuclear factor-E2 related factor 2 (Nrf2), a member of the Cap’n’ collar family of basic-region leucine-zipper proteins, is a master regulator of cytoprotective genes through its binding to the antioxidant defense element (ARE) located in the promoter region of these genes. The Nrf2 pathway is physiologically activated by the common mediators of the inflammatory pathway, such as oxidative stress and MAPKs (e.g., extracellular signal-regulated kinase [ERK], c-Jun N-terminal kinases [JNKs], and p38 MAPK), thereby suggesting a counter-regulatory mechanism to mitigate injury to the surrounding tissue during inflammation [127]. It has been suggested that cysteine residues in Keap1 are sensitive to an altered intracellular redox status, and ROS or electrophiles can disrupt the Keap1–Nrf2 complex via direct oxidation or covalent modification of thiol groups within Keap1. Furthermore, the Nrf2–Keap1 signaling can be modulated indirectly by the post-transcriptional modification of Nrf2. Phosphorylation of Nrf2 on its serine and threonine residues by several kinases such as MAPKs, protein kinase C (PKC), and phosphoinositol 3-kinase (PI3K) results in the activation of Nrf2 [128]. One explanation for the anti-inflammatory effect of Nrf2 is that the Nrf2-dependent induction of phase II genes suppresses the activation of the transcription factor NF-κB, thereby blocking the transcription of pro-inflammatory mediators and cytokines [129]. Along with the widely accepted viewpoints that Nrf2 prevents inflammation through the regulation of redox homeostasis, a recent study has shown that Nrf2 binds to the proximity of pro-inflammatory cytokine genes and inhibits RNA pol II recruitment, thereby blocking the transcriptional upregulation of these genes [130].

It can be surmised that a significant portion of the antioxidant and anti-inflammatory properties of tea polyphenols may be associated with the inhibition of the metabolic activation of carcinogens by phase I enzymes, coupled with the induction of phase II detoxification and antioxidant enzymes. It has been reported that tea polyphenols (200 mg/kg) suppress phase I carcinogen-activating enzymes such as CYPs and simultaneously upregulate the levels of phase II detoxifying and antioxidant enzymes. For example, in the tongue and oral cavity tissues of 4-nitroquinoline 1-oxide (4-NQO)-induced rat oral cancer, it was observed that both co-treatment and post-treatment with green tea polyphenols significantly reduced the activity of phase I enzymes, including cytochrome b5, CYP, cytochrome b5 reductase, aryl hydrocarbon hydroxylase, and DT-diaphorase, but elevated the activity of phase II detoxifying enzymes such as GST and UDP-GT [131]. That study proposed that green tea polyphenols acted as a potential chemopreventive agent that inhibits the bioactivation of 4-NQO and simultaneously enhanced the detoxification of metabolite carcinogens, thereby attenuating tumor development. Being an indirect carcinogen, DMBA undergoes metabolic conversion to produce electrophile metabolites by CYP monoxygenases, which are inhibited by the presence of green or black tea polyphenols (4 cups of teas equivalent to 30–40 mg of tea polyphenols/kg body weight by human) [132]. Another study reported that presupplementation with Polyphenon-B or BTF-35 significantly decreased DMBA-induced hamster oral tumorigenesis possibly by blocking the activity and expression of phase I enzymes—CYP 1A1 and CYP 1B1—and increasing the activity of phase II enzymes in the buccal pouch and liver [66]. In a mouse model of paracetamol-induced hepatotoxicity, it was observed that tea polyphenols (200 or 400 mg/kg/day) downregulated the expressions of CYP 2E1 and CYP 1A2, which catalyze the metabolism of paracetamol to a highly electrophilic N-acetyl-p-benzoquinoneimine (NAPQI), thereby preventing liver injury [133]. In a mouse model of restraint stress, it was found that increased levels of CYP 1A2, 2D22, 2E1, and 3A11 were normalized by EGCG pretreatment (40 mg/kg), thereby contributing to decreased stress response [134]. Theaflavins in black tea were found to suppress the omeprazole-induced expression and activity of cytochrome P450 (CYP) 1A1 in HepG2 cells. Likewise, both decaffeinated black tea extract and polymeric black tea polyphenols significantly decreased the activity and expression of benzo(a)pyrene-induced CYP 1A1 and CYP 1A2 in mouse livers and lungs [135].

Nrf2 is considered as a target for the prevention of diverse pathological conditions. Green tea polyphenols have been considered as inducers of the Nrf2-mediated antioxidant and detoxification pathway [136]. Both green tea polyphenols and individual green tea catechins were found to induce the expression of phase II detoxifying enzymes through ARE in HepG2 cells [137,138]. These upregulations were accompanied by the activation of MAPK, although catechins exerted distinct effects on MAPK; for instance, EGCG exhibited potent activation of all three MAPKs (ERK, JNK, and p38), whereas EGC activated ERK and p38. Another study demonstrated that treatment of human breast epithelial cells (MCF10A) with EGCG (100 µM) induced the Nrf2 pathway, as evident from increased nuclear accumulation, ARE binding, and Nrf2 transcriptional activity as well as increased expression of glutamate–cysteine ligase catalytic subunit (GCLC), MnSOD, and HO-1. In that study, EGCG treatment was found to remarkably activate the ERK1/2 and Akt signaling pathways [139]. Analysis of gene expression profiles by cDNA microarray and analysis of the 5′-flanking region showed that green tea extract (100 µg/mL) strongly induced the activity of phase II detoxification enzymes through the activation of ARE in HepG2 cells but not in the Cal-27 cell line [140]. Such activation was also observed in an in vivo study, wherein oral administration of EGCG (200 mg/kg) to C57BL/6J mice induced the expression of Nrf2-dependent genes such as GCLC, gamma-glutamyltransferase 1, aldehyde reductase-like 6, sialyltransferase 10 in the liver, and HO-1 in the small intestine [141].

A study reported that tea polyphenols exert neuroprotective effects against oxidative stress-induced apoptosis through the activation of the Keap1/Nrf2 antioxidant defense pathway in SH-SY5Y neuronal cells and mice brain. That study indicated that the PI3K/Akt signaling pathway was required for the tea polyphenol-mediated activation of Nrf2 pathway [142]. Moreover, tea polyphenols exerted hepatoprotective effects against oxidative stress by upregulating the activities of Keap1/Nrf2 pathway-mediated phase II detoxifying and antioxidant enzymes in HepG2 cells and mice liver [143]. In a model of fluoride-induced rat lung injury, pretreatment with EGCG was found to improve antioxidant status and inhibit oxidative stress as well as inflammation through the activation of the Nrf2 pathway. Furthermore, molecular docking analysis supported the antioxidant capacity and Nrf2 activation of EGCG by inhibiting the Keap1 repressor activity [39]. Using a model of mice irradiated with γ-ray (Gγ) total body irradiation (TBI), a study demonstrated that theaflavin reduced the TBI-induced oxidative stress in hematopoietic stem and progenitor cells (HSPCs) by upregulating the activities of Nrf2 and phase II detoxification and antioxidant enzymes; however, theaflavin failed to reduce the oxidative stress of hematopoietic stem cells in Nrf2^−/−^ mice [64]. This finding demonstrates the role of Nrf2 in the theaflavin-mediated antioxidant protection for irradiated HSPCs.

### 4.2. Inhibition of NF-κB and AP1 Pathways by Tea Polyphenols

**Figure 4:**
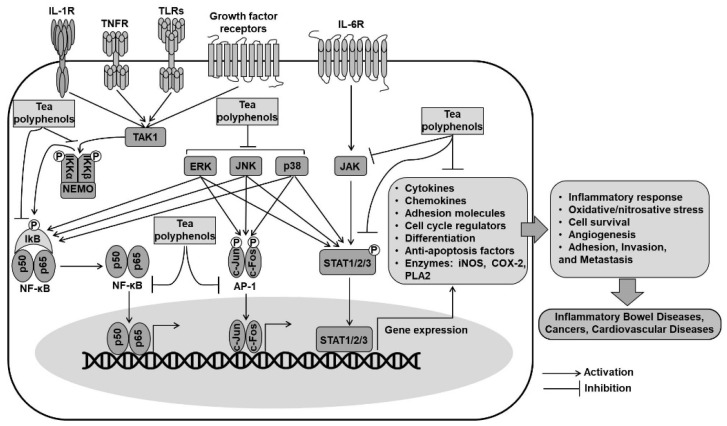
Cytoprotective mechanisms of tea polyphenols via regulation of inflammatory signaling pathways. AP-1, Activator protein 1; NF-κB, Nuclear factor kappa B; NEMO, NF-kappa-B essential modulator; STAT, Signal transducer and activator of transcription; ERK, Extracellular signal-regulated kinase; JNK, c-Jun N-terminal kinase; JAK, Janus kinases; IKKα/β, Inhibitor of nuclear factor Kappa-B kinase subunit alpha/beta; IκB, Inhibitor of κB; TAK1, Transforming growth factor-β-activated kinase 1; TLRs, Toll-like receptors; TNFR, Tumor necrosis factor receptor; IL-1R, Interleukin-1 receptor; IL-6R, Interleukin-6 receptor.

Nuclear factor-kappa B (NF-κB) and activator protein 1 (AP1) are redox-sensitive transcription factors that play a vital role in some signal transduction pathways involved in chronic inflammatory diseases and cancer (Figure 4). Various exogenous and endogenous factors such as pathogen-related molecules, cytokines, T-cell-activating signals, and reactive species provoke signal transduction cascades that rapidly activate NF-κB and AP1, which act independently or coordinately to regulate the expression of the target gene [144].

NF-κB is generally inactivated in the cytoplasm by binding to inhibitory kappa B (IκB). IκB becomes functionally inactivated and degraded by the phosphorylation of IκB kinases (IKKs), and subsequently, NF-κB is liberated from its association. Then, the released NF-κB is translocated into the nucleus, where it binds to specific promoters and transcriptionally promotes the expression of inflammatory genes, including iNOS, COX-2, cytokines, chemokines, and intercellular adhesion molecules [127,144]. AP1 is another transcription factor that regulates the expression of genes involved in transformation and proliferation. It is a complex of either homodimers or heterodimers of proteins of Jun (c-Jun, JunB, and JunD) and Fos (c-Fos, FosB, Fra-1, and Fra-2) families, which interact through a basic-region leucine-zipper domain [126,144]. AP1 can interact with other proteins, including the p65 subunit of NF-κB, to form novel protein complexes exhibiting enhanced biological functions [145,146]. Accumulating research shows that both NF-κB and AP-1 are regulated by the MAPK-signaling cascades.

The anti-inflammatory effects of tea polyphenols are associated with reducing the production of pro-inflammatory mediators and cytokines through the downregulation of NF-κB and AP-1 pathways. Studies have shown that EGCG suppressed the activity of IKKs concomitant with NF-κB inactivation in the intestinal epithelial cells [147], human osteosarcoma SAOS-2 cells [148], and human chondrocytes [149]. It has also been demonstrated that the EGCG-mediated inhibition of IKK activity appears to correlate with the presence of the gallate moiety in the C-ring because polyphenols lacking the gallate group were unlikely to inhibit IKK activity [147]. EGCG was also found to inhibit NF-κB activation by preventing the phosphorylation and subsequent degradation of IκB in respiratory epithelial cells [150] and human mast cells [151]. In general, tea polyphenols may inhibit NF-κB activation by the direct inhibition of IKK activity or by interfering with the interaction of IKKs with its target IκB [152]. Green and black tea polyphenols exhibited a similar inhibitory effect on the activity of AP1 and growth of JB6 cells transfected with a mutant H-ras gene. Comparisons among green tea catechins indicated that the presence of a galloyl structure in the B-ring or a gallate moiety exerted a higher suppressive effect on AP1 activity, and the galloyl structure appeared to possess a stronger activity than the gallate residue. Furthermore, the green tea polyphenol EGCG (20 µM) inhibited AP1 activation by decreasing the phosphorylation of c-Jun and ERK, whereas the black tea polyphenol TF3 (20 µM) reduced the levels of p-cJun, Fra-1, p-ERK, and p-p38 [153]. Structural and functional analyses from the above-described studies suggest that the gallate moiety and galloyl structure are responsible for the anti-inflammatory properties of tea polyphenols.

A study demonstrating the crosstalk between NF-κB and Nrf2 indicated that the activation of Nrf2/ARE-regulated antioxidant signaling was critical for the inhibition of the TLR4/NF-κB-mediated inflammatory response in LPS-induced RAW 264.7 cells [154]. Pretreatment with tea polyphenols (100 or 200 mg/kg for 10 days) to rats prevented renal ischemia–reperfusion injury by inhibiting the TLR4/NF-κB pathway and concurrently restoring the activity of endogenous antioxidant enzymes [155]. In the study on the gastric ulcerated mice, it was observed that black tea extract and theaflavins markedly decreased the serum levels of adhesion molecules (s-VCAM and s-ICAM) and Th1 pro-inflammatory cytokines (TNF-α and IL-6) but increased the serum levels of Th2 anti-inflammatory cytokines (IL-4 and IL-10). That study showed that the anti-inflammatory effects of black tea extract and theaflavins were more significantly effective compared with those of the positive control omeprazole [90].

### 4.3. Inhibition of the Signal Transducer and Activator of Transcription (STAT) Pathway by Tea Polyphenols

STAT proteins are cytoplasmic transcription factors that are activated by the phosphorylation of Janus kinases (JAKs) and other kinases in response to cytokines and growth factors (Figure 4). The activated proteins migrate into the nucleus and drive the transcription of genes related to inflammation such as iNOS, IL-6, and intracellular adhesion molecule 1 (ICAM-1). Increased expression and activation of STAT1 and STAT3 have been observed in patients with IBD, especially those with ulcerative colitis, suggesting their importance in the pathogenesis of colonic inflammation [156,157].

Tea polyphenols, including EGCG and theaflavins, were found to inhibit the UVB-induced STAT1 activation by blocking the ERK, JNK, PDK1, and p90RSK2 signaling pathways [158]. The EGCG-mediated inhibition of STAT1 activation exhibited beneficial effects with respect to inflammation [159] and epithelial barrier function [160] through the modulation of its response to interferon gamma (IFNγ). Moreover, EGCG might prevent STAT1 activation by blocking the phosphorylation of upstream kinases [161] or by directly binding to the STAT1 protein [162]. Pretreatment of mice with green tea polyphenol extract (25 mg/kg i.p for 1 h) was found to attenuate the carrageenan-induced DNA-binding activity of STAT1 and lung injury [163]. EGCG supplementation (1% EGCG in diet) was also found to attenuate obesity-associated neuroinflammation and microglial activation by suppressing the JAK2/STAT3 signaling pathway in both cellular and high-fat-diet rodent models [164].

## 5. Conclusions

Tea and its products are sources of antioxidant polyphenols with diverse classes of polyphenolic compounds. Epidemiological studies suggest the benefits of tea polyphenols in preventing chronic diseases and promoting physiological functions. Tea polyphenols act as direct and indirect antioxidants that help attenuate oxidative/nitrosative stress and prevent cells/tissues from oxidative damage. Evidence shows that tea polyphenols could act as chemopreventive agents that eliminate reactive intermediates and carcinogens before they can react with DNA, accounting for a lower risk of developing cancer. Furthermore, the health-promoting effects of tea polyphenols are due to their ability to modulate cellular signaling transduction pathways. This review has partially shed light on the relationship between specific chemical structures of tea polyphenols and their biological activities. However, further investigation is required to evaluate the therapeutic roles of tea polyphenol as well as the relevant mechanisms and establish the safe range of tea consumption associated with the benefits.

## Data Availability

Not applicable.
